# Combustion Behaviors of CIGS Thin-Film Solar Modules from Cone Calorimeter Tests

**DOI:** 10.3390/ma11081353

**Published:** 2018-08-04

**Authors:** Lulu Yin, Yong Jiang, Rong Qiu

**Affiliations:** State Key Laboratory of Fire Science, University of Science and Technology of China, Hefei 230026, China; ycode@mail.ustc.edu.cn (L.Y.); rqh@mail.ustc.edu.cn (R.Q.)

**Keywords:** photovoltaic fires, CIGS, flammability, fire hazard, cone calorimeter

## Abstract

As is well known, building integrated photovoltaic (BIPV) technology is becoming more commonly used in residential and commercial buildings. Fire assessment of photovoltaic (PV) modules as a whole is still insufficient. This work focuses on the thermal properties and combustion behavior of CIGS (copper, indium, gallium and selenium) thin-film modules. Cone calorimeter experiments were conducted at different external heat flux of 25, 30, 35, 40 and 45 kW m^−2^. Several parameters are discussed, including surface temperature, ignition time, heat release rate (HRR), mass loss rate, carbon monoxide (CO) and carbon dioxide (CO_2_) concentrations. The results show that CIGS thin-film solar modules are inflammable at intermediate or high flashover risk. A correction calculation for the gas toxicity index has been used to reduce the well-ventilation condition effect. Compared with the uncorrected calculation, peak fractional effective dose (FED) and lethal concentration for 50% of the population (*LC*_50_) are almost double. This work will help to determine a more stringent fire safety provision for PV modules.

## 1. Introduction

### 1.1. Background

Solar cells have been used in building integrated photovoltaic (BIPV) systems, vehicles, aerospace applications and solar power plants. Photovoltaic (PV) technologies are divided into three generations, which are wafer-based (1st generation PV), thin-film cell (2nd generation PV) and new emerging technologies (3rd generation PV). However, the latter has not been used in the PV market recently [1]. The advantages of thin-film solar technologies are flexibility and minimum material usage for good cost effectiveness [2]. Furthermore, the conservation of energy, materials and thin-film processes are eco-friendly.

As for PV device design and fabrication, there are choices to be made in areas such as substrates (flexible or grid, metal or insulator), layers (e.g., contact, buffer, absorber reflector, etc.) and techniques (e.g., PVD, CVD, ECD, plasma-based, hybrid, etc.) [3]. The most widely commercialized thin-film solar cells include a-Si (thin-film amorphous silicon), CdTe (compound semiconductor cadmium telluride) and compound semiconductor made of CIGS (copper, indium, gallium and selenium). CIGS solar cell is presently regarded as the highest light-to-power conversion efficiency material [2,4]. The best efficiency of CIGS is 22.3% [5], compared with CdTe (22.1%) [6] and α-Si (13.6%) [7].

New techniques such as nanotechnology improve solar cell application efficiency. Adding reduced erbium-doped ceria nanoparticles as a coating on silicon solar cells can improve efficiency from 15% to 16.5% [8]. For CIGS cells, random distribution of Au and Ag nanoparticles broadens the resonance wavelength of the transmittance, and improves efficiency by 1.2% and 1.4% [9]. Hyper branched nanostructures also increase efficiency. When it used in FTO-glass sensitized with D-102 dye, a maximum efficiency of 3.96% is reached [10]. Al-doped ZnO nanostructured films as transparent electrodes in photovoltaic devices shows high Haze factor (>80%) and may be exploited to enhance the light trapping capability [11]. Metallic nanowires could boost the conductivity of the front conductor, while the optical penalty can be solved by introducing a texture [12]. Thin-film techniques make solar modules become a promising approach for terrestrial and space applications. Thin-film modules are a suitable choice for new building types, for instance ETFE (ethylene tetrafluoroethylene) cushion structure. ETFE structure is often used in stadiums and airport terminals, like the National Aquatics Center for the 2008 Olympic Games. Temperature distribution and characteristics of a two-layer ETFE cushion integrated flexible PV also had been studied in Hu’s research [8].

These technologies ensure CIGS thin-film solar modules’ mass production and prospects for extensive use. Modules used in our test are CIGS thin-film PV with a cover layer of ETFE. This module can be integrated on curved surfaces due to its flexible substrate. Samples used in our research are made of transparent contact-layer, blue cell, green cell, red cell, reflecting metal-layer and flexible substrate [13]. Compared with Si PV, they have light-spectrum-splitting capacity to reach higher efficiency [14].

### 1.2. Fire Hazard

PV system fires are common in residential and commercial properties. PV systems are often accompanied by high life-threatening voltages, from 300 to 1000 Volts DC [15], and have the potential risk of spontaneous ignition. When a fire occurs, it is hard to cut off the electric circuit to make sure all components are de-energized. The light-to-power systems work under light irradiation as long as they are not totally destroyed. For firefighters, it is much more difficult to deal with the potential high voltage, since it puts their lives in danger. At present, PV module research has mainly focused on fire-resistance testing and fire preventing. There are several standards for PV module fire safety tests, such as IEC 61730-2 [16], UL 1703 [17] or even UL 1256 [18]. New research still improves these standards. For example, a modified IEC 61730-2 [16] by Wohlgemuth et al. [15] includes overheat caused by hot spots, high series resistance or arcing.

It is worth doing research about the burning behaviors and fire risks of solar modules. Guerin confirmed the risk of fire with the large-scale solar photovoltaic construction project in Reference [19]. Based on the perspective of firefighters, Casey focused on firefighting of solar photovoltaic panels. Casey also suggested a practice guidance for firefighters for emergency response [20]. Guerin and Casey notice that the performance of rooftop solar panels under radiant heat is unknown. Besides this, computational fluid dynamics (CFD) fire modeling tools also need effective material properties, especially for large-scale simulation. Thermal degradation of solid fuels in a fire situation is complex, because of interactions between different materials. Taking PV modules as a whole is a direct way to reduce the amount of computation required.

However, fire hazard research concerned with taking PV modules as a whole is still insufficient. In order to investigate the emissions and redistribution of elements, a commercial CdTe PV module was heated up to 1273 K to simulate exposure to fires [21]. Yang et al. focused on the flammability and fire hazards of polycrystalline silicon PV modules with glass covering [22]. In their research, a whole silicon PV module was ignited under external radiation during cone calorimeter tests. Both heat and smoke were discussed, because those are necessary when assessing full-scale fire. Cone calorimeter is a widely used device to measure fire reaction properties, in fire potential assessment of wood, polymer, and even for batteries. Fu’s paper concerning cone calorimeter tests of lithium ion batteries indicates that the collected data can be used directly, as well as input data for mathematical models to analyze the thermal and chemical threats [23]. 

Our research focuses on the combustion behaviors and thermal hazards of CIGS thin-film solar modules. We also discuss the difference of fire behaviors, compared to Yang’s results for rigid polycrystalline silicon PV modules. Compared with rigid solar modules, flexible modules use polymer as the top layer and flex backsheet as bottom layer. Additionally, encapsulant layers bring more combustible. In order to evaluate the effects of irradiation on properties and discuss the thermal properties and gas toxicity, these parameters are measured: surface temperature, ignition time, heat release rate, mass loss rate, CO concentration and CO_2_ concentration. An evaluation system proposed by Petrella has been introduced to classify the danger of heat contribution and flashover quantitatively. Well-ventilation cone calorimeter test condition causes an underestimate of toxic gas concentration. This underestimate could be dangerous when assessing building fire safety level and arranging for evacuation. Han and Chow provide a correction calculation for the gas toxicity index in cone calorimeter test [24]. In our work, we adopt these two evaluation systems and compare the results of Petrella’s and Han and Chow’s.

## 2. Experimental Setup

### 2.1. Samples

**Figure 1:**
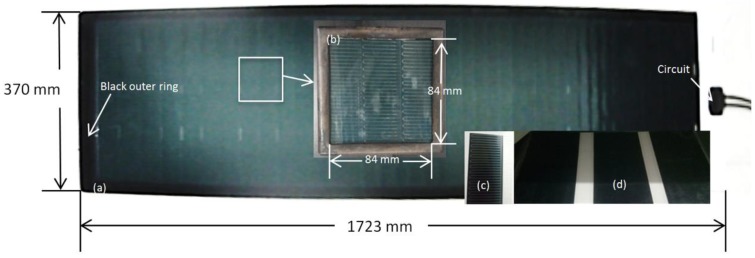
Copper, indium, gallium and selenium (CIGS) thin-film solar module and sample. (**a**) module; (**b**) specimen; (**c**) CIGS solar cell sample; (**d**) adhesive.

In this experiment, samples were collected from MiaSolé FLEX-01 70N [25]. FLEX-01 70N is a manufactured product with a scale of 1723 mm × 370 mm × 2.5 mm (thickness with adhesive), shown in Figure 1a. This batch production can be separated into three parts: sensitive part, back adhesive and periphery. The sensitive part is the section with CIGS solar cells, which is the functional area that transforms light energy to electrical energy. Figure 1b shows the sensitive part as a specimen in sample holder. Figure 1c shows a slice of packaged CIGS solar cell, with the sensitive part having a width of 5 cm. Back adhesive helps the module to remain fixed and does not have a light to electric transformative function, shown in Figure 1d. There are three adhesive slices, with a width of 10.3 cm each. On the periphery is the outer edge of the sensitive part, with the function of protection and electric circuit. As shown in Figure 1, the black outer ring (with a width of 2.2 cm) around the module is the periphery to protect the sensitive parts. Only the sensitive part was taken into consideration, without back adhesives and periphery. Samples were of the size 100 mm × 100 mm × 1.5 mm with a weight of 29 ± 2 g. All samples were wrapped using the shiny side of an aluminum foil layer. Then samples were put in a holder frame with an open window area of 84 mm × 84 mm. The sample can be separated into the four layers of ETFE, solar cells, metal backboard, and polyethylene terephthalate (PET), from top to bottom.

### 2.2. Apparatus

**Figure 2:**
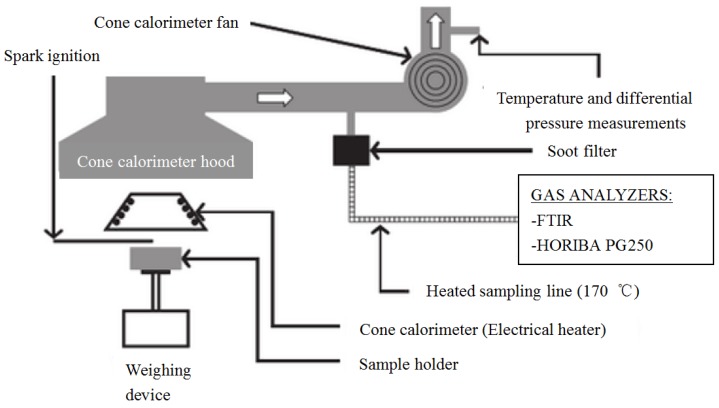
Schematic of a cone calorimeter [27].

Experiment procedures were conducted according to the ISO 5660 standard [26] with a cone calorimeter developed by Fire Testing Technology under well-ventilated conditions. The schematic of a cone calorimeter is shown in Figure 2 [27]. Forced-flaming combustion by external radiation was chosen to investigate flame. Tests were conducted at various levels of heat flux ranging from 20 to 45 kW m^−2^. In order to reach real fire heat flux level, external radiation was employed. Babrauskas suggests that 25–50 kW m^−2^ is suitable for most research purposes [28]. In this research, 45 kW m^−2^ was enough to reach a short enough ignition time. The experiments were stopped manually according to the mass loss rate criterion. That means the test ends when the average mass loss rate drops lower than 1 g m^−2^ in a 60 s period. Two K-type thermocouples were used in the thermally thick tests to get the temperature of upper and lower surfaces. For each condition, tests were repeated at least twice to ensure reproducibility. These parameters were measured: surface temperature, ignition time, heat release rate, mass loss rate, CO concentration and CO_2_ concentration.

## 3. Results

### 3.1. Burning Behavior

**Figure 3:**
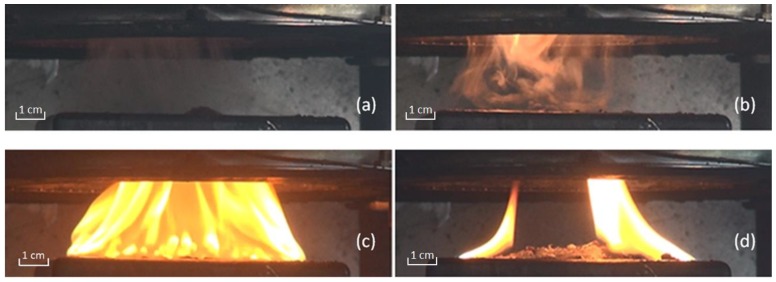
Burning process of CIGS specimen. (**a**) incipient stage; (**b**) ignition; (**c**) fully burning stage; (**d**) extinction stage.

Figure 3 presents the screenshots from a combustion test video shot using a Canon digital video camera. It presents a burning progress with the four typical stages of heating, ignition, rapid burning and extinction. After being exposed to external heat flux for a while, vapors rise above the sample surface. Then vapors increases in quantity. The front ETFE cover melts with blistering, shown in Figure 3a. With high external radiation, the blistering is more intense. Then ignition appears from the higher part of the steam, shown in Figure 3b. Flammable steam is lit, and the combustion begins. This time interval from exposed to external radiation to the ignition is usually called ignition time ($t_{ig}$). With higher external radiation, ignition comes earlier. The flames become larger rapidly with a cluster of bubbles, shown in Figure 3c. The fire reaches its maximum and then becomes smaller gradually. With the fuel running out, the flames extinguish, shown in Figure 3d. After the test, solid residue can be found in the sample holder, such as burning ash, CIGS cells, metal etc. During the heating period, shape changes of the three CIGS chips happened, while they used to be arranged in parallel. These shape changes lead to lower-layer combustible melts exposure to the fire, air and external heat flux. In this experiment, the sample holder can be considered as a limited space without combustible melts spreading out. In a real fire, these melts can exacerbate the risk of a fire spreading.

**Figure 4:**
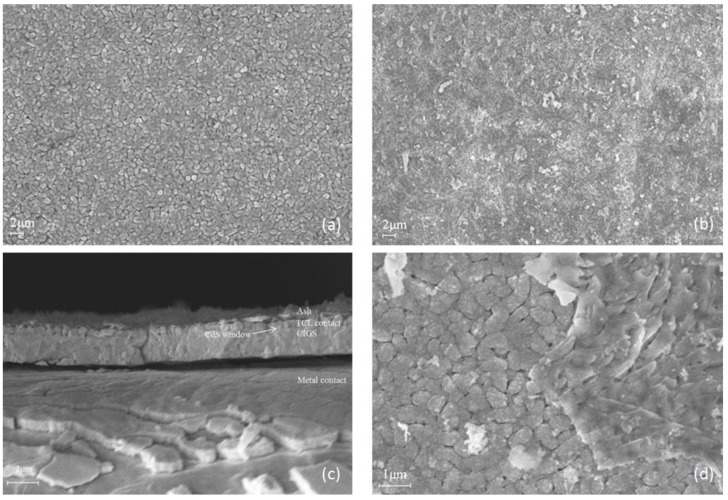
SEM images of CIGS cell layer. (**a**) top surface image before burning; (**b**) top surface image after burning; (**c**) cross section image after burning; (**d**) details of ash attachment.

Figure 4 shows the SEM images of CIGS cell layer. The device used is a GeminiSEM 500, Carl Zeiss, Germany. Figure 4a,b shows the top surface before and after burning test under external heat flux of 40 kW m^−2^. Before the test, uniform size particles can be found on the surface. After the test, the surface becomes irregular. Figure 4c shows the cross section image after test. A typical structure of CIGS thin-film solar cell can be separated into five layers: transparent conductive layers (TCL), CdS window, CIGS absorber, metal contact and substrate, from top to bottom [29]. These five layers can be found in Figure 4c. TCL are usually thin conductive metal oxides. MiaSolé used ZnO for their front contact and Mo for their back contact [30]. Figure 4d shows the details. It shows that surface particles do not melt or disappear. Some attachments appear on the surface and cover the TCL contact. Attachments may be the burning residue of ETFE.

### 3.2. Thermally Thick

Determining the CIGS module sample as thermally thin or thick is the premise to the parameters of ignitability and combustibility. Biot number (Bi) is calculated to classify whether material is thermally thick or thin [31], and can be expressed as:

(1) $$Bi=\frac{hL}{k}\;$$

where h is heat transfer coefficient, L is characteristic thickness, and k is thermal conductivity of the solid, respectively. However, for a multi-material sample, it is hard to use this equation. An experimental method is presented below. With a thermally thick solid, the gradient of temperature in the solid sample can be observed [32]. Because of this feature, a temperature difference test was performed as follows, also used in Reference [33].

**Figure 5:**
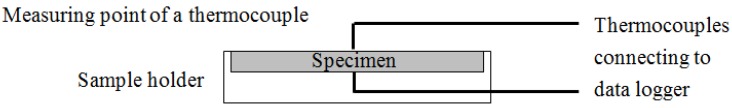
The schematic of setting up thermocouples.

**Figure 6:**
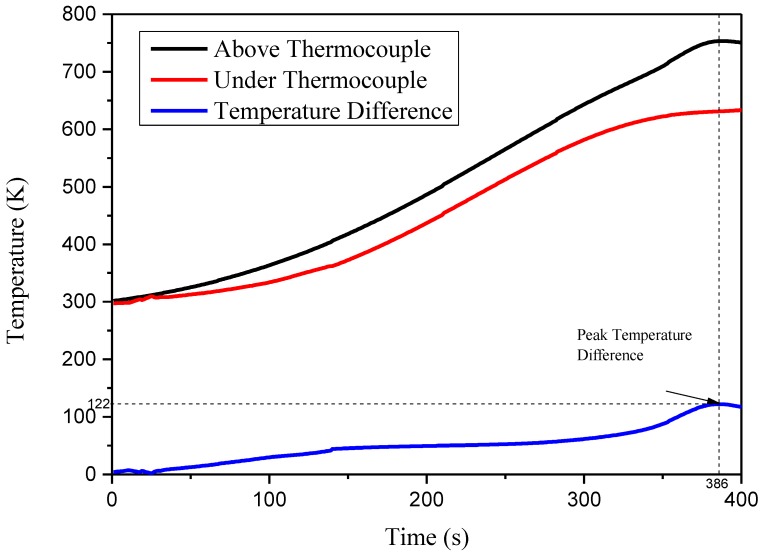
Temperature-time curves for two thermocouples at 20 kW m^−2^.

Thermocouples were placed at the upper and lower surfaces of the specimen to monitor the temperature difference, shown in Figure 5. The specimen was exposed to heat flux of 20 kW m^−2^. In the pre-test, specimens could not ignite at less than 20 kW m^−2^. This value is suitable for the temperature tests, because flame interference is eliminated. Temperature conducted by the thermocouple is shown in Figure 6. Temperature difference can be observed between two thermocouples during the whole test. The peak temperature difference is 122 K. Temperature difference indicates that heat conduction inside the sample is much slower than heat convection away from its surface. Thus, the sample is thermally thick.

### 3.3. Ignition Time

Ignition time is a key parameter for fire resistance, and is an indicator of fire starting. Higher ignition time indicates longer time to heat up to ignition. The ignition time can be determined by the time interval from the initial exposure to irradiation (*t* = 0) to the moment a flame arises on the material surface.

A rapid decrease in ignition time is caused by the increase in heat flux. For example, $t_{ig}$ decreases from 128 s to 38 s when the heat flux increases from 25 to 45 kW m^−2^. Several simplified heat conduction models have been developed for further analysis [34,35,36]. The model used in this work is proved by Quintiere [37]. Sample ignition can be achieved when an external heat flux (${\dot{q}}_{e}^{\prime \prime }$) is higher than a critical heat flux (CHF). During a time interval ($t_{ig}$), the specimen ignites. For thermally thick materials, the ignition time is calculated by Equation (2):

(2) $$t_{ig}=\frac{\pi }{4}k\rho C_{p}{\left(\frac{T_{ig}-T_{0}}{{\dot{q}}_{e}^{\prime \prime }}\right)}^{2}\;$$

where ρ is density, $C_{p}$ is ignition constant, $T_{ig}$ is ignition temperature, $T_{0}$ is initial temperature, and ${\dot{q}}_{e}^{\prime \prime }$ is external heat flux. This equation is for thermally thick materials, also used in References [33,35,36,37]. In this model, the thermal response parameter (TRP) also has been computed, as Equation (3):

(3) $$\sqrt{\frac{1}{t_{ig}}}=\frac{\sqrt{4/\pi (q_{e}^{\prime \prime }-CHF)}}{TRP}\;$$

According to Equations (2) and (3):

(4) $$CHF=-\frac{{}_{{TRP\cdot y}_{\mathrm{int}ercept}}}{\sqrt{4/\pi }}\;$$

(5) $$TRP=\sqrt{\frac{4}{\pi }}\frac{1}{Slope}\;$$

**Figure 7:**
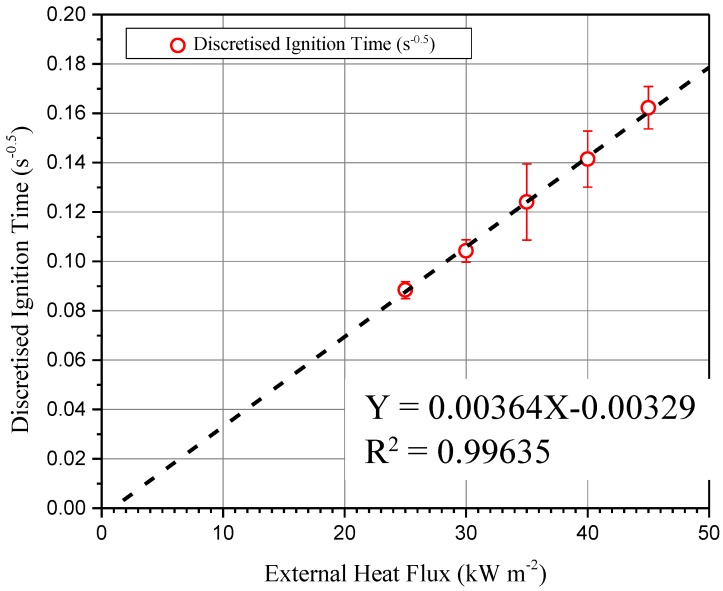
Square root of the inverse of ignition time (s^−0.5^) as a function of radiant heat flux (kW m^−2^).

According to Equation (3), the square root of the inverse of time of ignition is a linear correlation with irradiation, shown in Figure 6. The linear trend shows that the specimen is thermally thick. This result is consistent with the previous thermocouples test results in Section 3.2. The straight line fits the data with the slope = 0.00364 and the *y_intercept_* = −0.00329. By applying Equations (4) and (5) to the last results from Figure 7, we get CHF = 0.90385 kW m^−2^ and TRP = 309.9943 kW s^1/2^ m^−2^. Then we get the theoretical CHF. According to the physical meaning of CHF, ignition occurs when external heat flux is larger than CHF. It is noteworthy that no naked fire can be observed during 1000 s under external heat flux at 20 kW m^−2^. The CHF of ETFE in communications cable insulation and jackets is 22 kW m^−2^ [38]. Using the theoretical CHF brings an underestimation of the module’s retardant performance. The differences between experimental and extrapolated CHF are due to the following three reasons. Non-linearity of the ignition time leads to the non-linearity of the square root of the inverse of the ignition time for the lowest external heat flux, especially near the CHF [39]. Second, as a multi-layer sample, the cover layer also brings non-linearity [40]. Third, Delichatsios et al. [41] suggest that surface reradiation causes a higher critical heat flux than the extrapolated value.

### 3.4. Heat Release Rate

Heat release rate (HRR) is the rate at which fire releases energy, as discussed in many papers. HRR is considered to be the most important factor in controlling fire hazards, owing to its strong connections with several fire reaction properties [23]. It determines whether a neighbor module can be ignited when fire happens in a large solar power installation [42]. HRR also provides a correspondance between fire intensity and fire spread [43].

**Figure 8:**
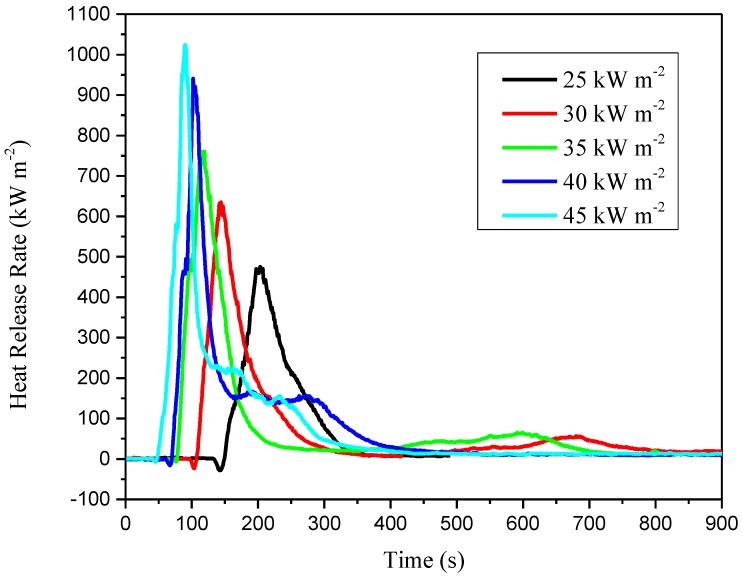
Heat release rate (KW m^−2^) versus time (s).

After a short time interval, HRR reaches its maximum, as peak HRR (pkHRR), and then it drops down to almost zero, as shown in Figure 8. When the heat flux increases from 25 kW m^−2^ to 45 kW m^−2^, the pkHRR changes from 475 kW m^−2^ to 1024 kW m^−2^. Furthermore, the time to reach pkHRR declines from 203 s to 90 s. 

In order to analyze the risks of heat contribution and flashover, Petrella’s evaluation system [43] is used, as with References [22,44]. In this system, two important parameters are proposed, called total heat release (THR) and *x* parameter. THR is the time integration of HRR, indicating the fire thermal hazards of the material, and is calculated using Equation (5):

(6) THR=∫HRR 

Additionally, the *x* parameter is calculated using Equation (3), as a fraction of the heat release rate peak (pkHRR) to ignition time ($t_{ig}$):

(7) $$x=\frac{pkHRR}{t_{ig}}\;$$

Table 1 shows the details of Petrella’s evaluation system.

From the experimental results shown in Table 2, and through comparison with Perella’s value, we arrive at the following conclusions:

(1) In the experimental range of external heat flux under 45 kW m^−2^, the CIGS thin-film solar cells have intermediate low risk to heat contribution.
(2) The flashover risk is intermediate when the external heat flux is 25 and 30 kW m^−2^.
(3) When external heat flux becomes more than 35 kW m^−2^, the risk to flashover turns to high, which is much more dangerous than polycrystalline silicon modules, according to Yang’s study [22].
(4) For a CIGS module used in the research with the power of 70 W, it releases energy of 49.49 MJ with the surface area of 0.638 m^2^ with external heat flux of 45 kW m^−2^. Furthermore, BIPV systems always need multiple slices to form a panel, and even more for an array. Large-scale usage of this module, especially on the roof or wall of high-rise buildings, brings heavy fire load.

### 3.5. Mass Loss Rate

**Figure 9:**
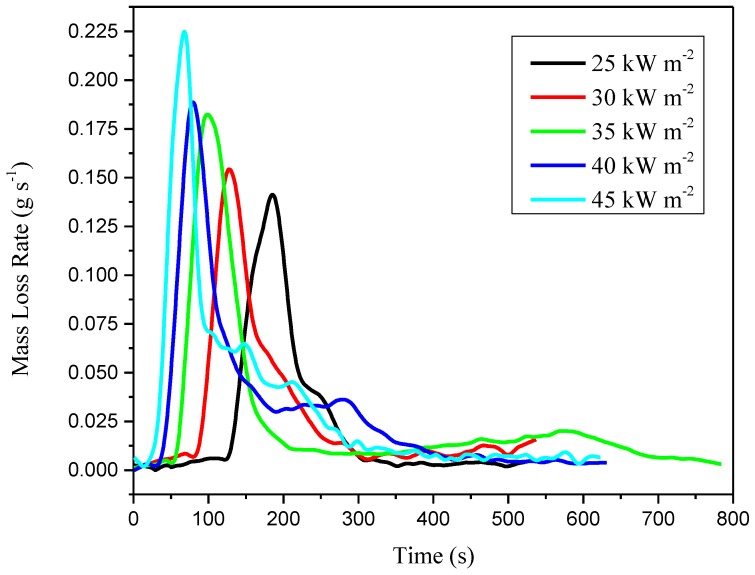
Mass loss rate (g s^−1^) as a function of experimental time(s).

The index to measure the level of pyrolysis, volatilization, and burning of the specimen during the whole cone calorimeter test under constant external heat flux is usually called mass loss rate (MLR). To calculate MLR, we chose five-point numerical differentiation equations. MLR is connected with heat release rate, specific extinction area, and CO yield [45], as shown in Figure 9. Mass loss rate curves reach their maximum values quickly with the increases of external heat flux, as the peak value at 25 kW m^−2^ is 0.225 g s^−1^.

Figure 8 shows the mass loss evolution and rate under different external heat flux. Data recorded during the experiment is shown, while time *t* = 0 means the moment of the exposure to the desired irradiance. The end time is after the extinguishment.

Regardless of different external heat flux, two stages of thermal degradation can be observed. The MLR curve shapes depend on the irradiance level value weakly, but the peak values are strongly related to radiation intensity. When external heat flux increases from 25 to 45 kW m^−2^, the time to reach the maximum becomes shorter. For example, this time interval shortens from 185 s at 25 kW m^−2^ to 68 s at 45 kW m^−2^. Additionally, a higher maximal intensity of the MLR peak is reached. It increases from 0.141 g s^−1^ at 25 kW m^−2^ to 0.225 g s^−1^ at 45 kW m^−2^. After a period of time exposed to heat flux, the sample ignites and MLR quickly rises. Then MLR reaches its peak value, then decreases. During the whole decomposition process under each radiation, only one MLR peak is observed. That means the composites keep their thermally thick properties. 

The specific mass loss rate (SMLR) is determined as a ratio between MLR and the exposed sample surface in the cone calorimeter. Moreover, SMLR can be calculated with Equations (8) and (9):

(8) $$SMLR=(\frac{1}{\Delta H_{g}}){\dot{q}}_{e}^{\prime \prime }+\frac{FHF-\varepsilon \sigma T_{ig}^{4}}{\Delta H_{g}}\;$$

(9) $$FHF_{net}=FHF-\varepsilon \sigma T_{ig}^{4}\;$$

where $\varepsilon \sigma T_{ig}^{4}$ radiative heat flux loss and $\Delta H_{g}$ is latent heat of gasification. FHF_net_ means the heat flux which the specimen contributes, as a minus of the flame heat flux sum and radiative heat flux loss from the sample surface [46,47].

**Figure 10:**
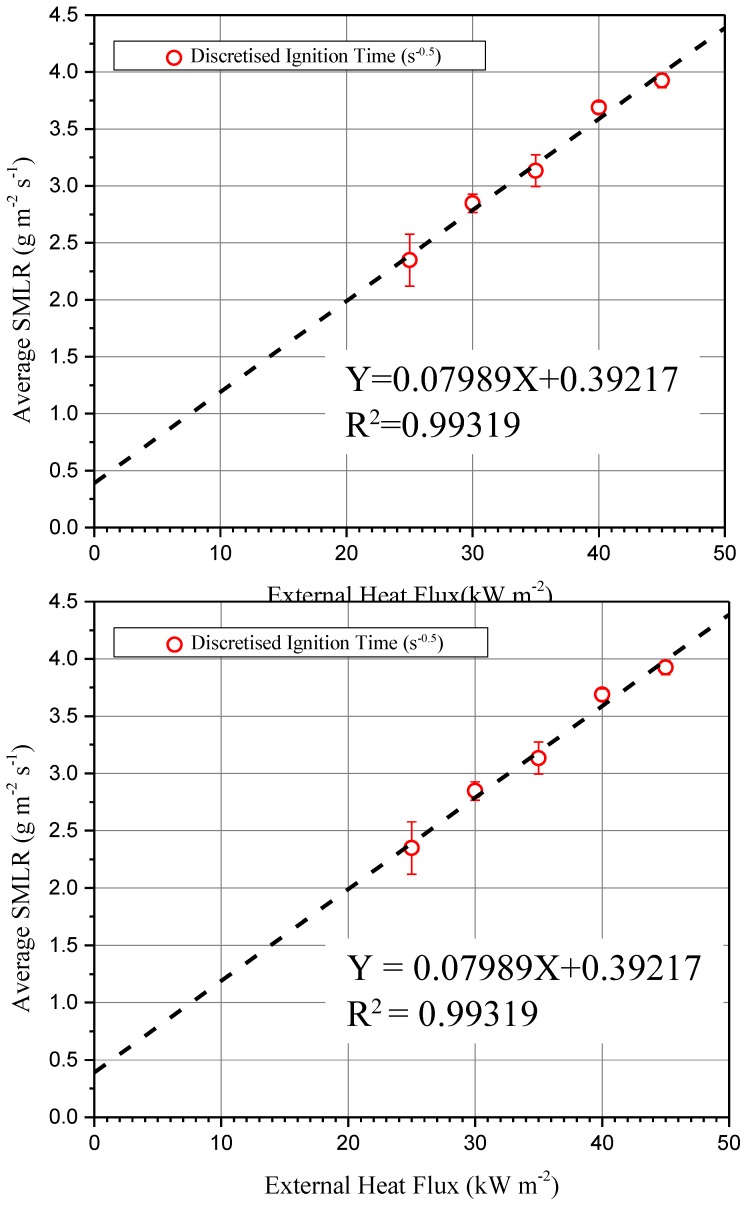
Averaged specific mass loss rate (g m^−2^ s^−1^) as a function of heat flux (kW m^−2^).

Figure 10 shows the average SMLRs increase quickly with the rise of the external heat flux. The average SMLR increases from 2.35 to 3.92 g m^−2^ s^−1^ when incident heat flux increase from 25 to 45 kW m^−2^. The linear trend of average SMLR curve shows that the heat of gasification changes little when the module is considered as a whole. According to Equation (8), the slope (=0.08) and *y_intercept_* (=0.39) of the best fit line of average SMLR vs. ${\dot{q}}_{e}^{\prime \prime }$ allow the computation of other thermal properties as Equation (10), such as gasification heat ($\Delta H_{g}$ = 12.52 kJ g^−1^), and net flame heat flux (FHF_net_ = 4.91 kW m^−2^). These two parameters are required in fire modeling. The gasification heat is also used to estimate the fire resistance of a material [32].

(10) $$Slope=\frac{1}{\Delta H_{g}};y_{\mathrm{int}ercept}=\frac{FHF-\varepsilon \sigma T_{ig}^{4}}{\Delta H_{g}}\;$$

### 3.6. Gas Toxicity

**Figure 11:**
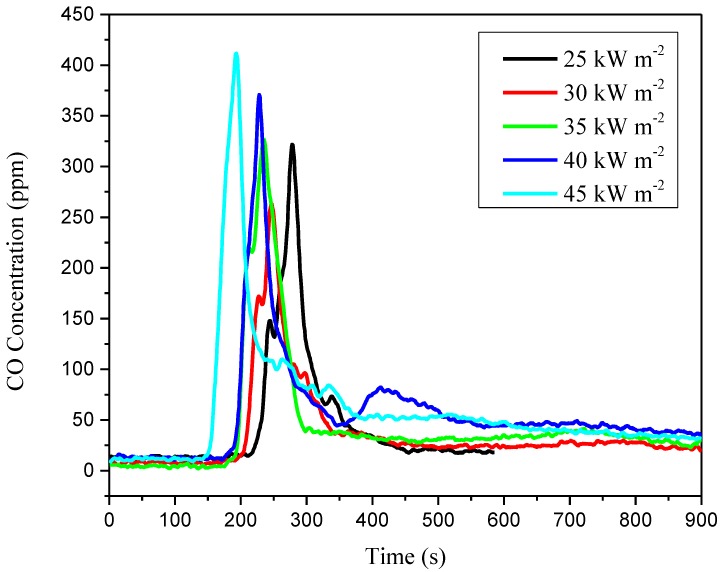
CO concentration versus time.

**Figure 12:**
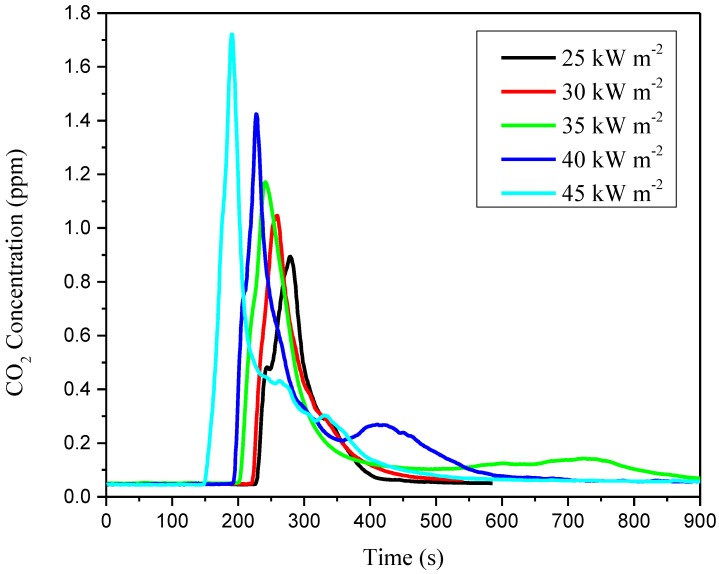
CO_2_ concentration versus time.

Smoke inhalation accounts for roughly three quarters of all fire deaths. The concentrations of CO and CO_2_ detected by the cone calorimeter are shown in Figure 11 and Figure 12. The CO_2_ and CO yields for lower external heat flux are lower than that for higher external heat flux. In particular, in the test of external heat flux at 45 kW m^−2^, the concentration of CO increased significantly after the ignition, and the concentration reached its maximum of 411.5 ppm with an aiguille on the curve.

More detailed experimental and derived data can be found in Table 3. It is observed that the maximum of concentration of CO_2_ increases from 0.89% to 1.72% while the external heat flux varies from 25 to 45 kW m^−2^. However, the concentration maximum of CO in ppm is not simply increased. When the external heat flux decreases, the concentration of CO decreases as well, but in the test of 25 kW m^−2^ (321.8 ppm), the concentration reaches a large value compared with that of 30 kW m^−2^ (263.4 ppm). As the CIGS thin-film solar cells’ special multi-layer structure, the stainless steel substrate on which the CIGS cells lie, and even the photovoltaic foils, brings difficulties for the under layers to contact air, especially when the external heat flux is not large enough to burn through the stainless steel substrate. The residue of the test of 25 kW m^−2^ found the stainless steel substrate with almost no damage, which signifies an incomplete combustion of the substrate. Thus, combustion may occur with more CO release.

The peak fractional effective dose (FED) is denoted by Equation (10) referring to the N-GAS model, which means the sum of the fraction of the concentration and the lethal concentration for 50% of the population (*LC*_50_) for each gas over a 30 min exposure time, with a 14-day post-exposure period, predicts that the fire gas will be lethal to 50% of a laboratory rat population [48].

(11) $$FED=\frac{m[CO]}{[CO_{2}]-b}+\frac{21-[O_{2}]}{21-LC_{50,O_{2}}}+\frac{\left[HCN\right]}{LC_{50,HCN}}+\cdots \;$$

Due to the fact that only concentrations of CO and CO_2_ are detected, FED is calculated from the peak concentration of CO and CO_2_ denoted by [CO] and [CO_2_] and the *LC*_50_ denoted by *LC_CO_* and *LC_CO2_*:

(12) $$FED=\frac{\left[CO\right]}{LC_{CO}}+\frac{\left[{CO}_{2}\right]}{LC_{{CO}_{2}}}\;$$

The toxic potencies of CO_2_ are very large, and FED can be calculated only from the peak value of [CO], denoted by pk[CO], taking the toxic potency *LC*_50_ of CO as 5000 ppm [44]:

(13) $$FED=\frac{pk[CO]}{5000}\;$$

It was found from a developed database that *LC*_50_ in actual fires would not deviate much from *LC*_50_ determined by bench-scale tests. However, Han and Chow suggest that the calculation of FED and *LC*_50_ in a cone calorimeter test is under well-ventilated conditions that may bring underestimation of the gas concentration [24]. They suggest an adjustment of gas concentration extracted from the burning facility with over-ventilated conditions, and this leads to a different result of FED and *LC*_50_. *FED* calculated in this way is denoted here as FED_cor_.

It is assumed that all the toxic gases can be collected in a chamber volume (*V_c_*) of burning air, the gas concentration increasing to the peak value at the time of burning out [24]. The *LC*_50_ (in ppmv or g m^−3^) is calculated from the sample mass loss Δ*m* and *V_c_*:

(14) $$LC_{50}=\frac{\Delta m}{FED_{cor}\times V_{c}}\;$$

In order to get the FED_cor_, a transient concentration of CO as a volume ratio, *[CO]_t_*, is given by the ratio of the integrated volume $V_{CO}^{0\to t}$ and *V_c_*:

(15) $${\left[CO\right]}_{t}=\frac{V_{CO}^{0\to t}}{V_{c}}=\frac{\int_{0}^{t}{\left[CO\right]}_{Cone}{\dot{V}}_{Cone}dt}{V_{c}}\;$$

The results of FED_cor_ and *LC*_50_ are shown in Table 3, with the volume *V_c_* setting to be 0.01 m^3^ like [25].

The data shown in Table 3 brings us to the following conclusions:

(1) Compared with values without the consideration of good ventilation, FED_cor_ deduced using Han and Chow’s method is almost twice as high. *LC*_50_ is 26.72 g m^−3^ at 30 kW m^−2^, and is higher than that in other conditions.
(2) Calculating FED is an intermediate step to deduce *LC*_50_. A proper way to get *LC*_50_ from cone calorimeter tests is relatively simple and easy to operate.

## 4. Discussion

Thermal properties and combustion behavior of CIGS thin-film solar modules was studied in detailed. CIGS thin-film solar cell technology is considered to be a promising substitute for fossil fuel because of its high efficiency and mass manufacture. Solar modules have been used as a part of exterior wall covering in personnel-intensive area. Under the premise of whole-module testing, a series of bench-scale tests on a cone calorimeter test bed were conducted. The results are compared with the glass covered polycrystalline silicon PV modules by Yang [22].

Taking MiaSolé as a sample, the same flexible cells are used for both glass and flexible modules [49]. However, the module structures are completely different. A glass module can be separated into five layers: top glass, encapsulant, cells, encapsulant and back glass. The main combustible component is encapsulant. As for a flexible module, the front and back barriers change from glass to polymer. These changes bring more combustible components.

In the incipient ignition stage, ignition behavior is different. In Yang’s research, fire ignited from the edge of Si PV specimen. This fire behavior mainly results from the glass cover layer of module. As a non-combustible material, glass covering used in a PV module also has better fire-resistance performance. It is good at resistance to fire penetration and transfer of excessive heat. Under the glass cover, some combustible materials, like encapsulate (i.e., ethylene-vinyl acetate copolymer) are much easier to ignite at the specimen’s verge. This is because encapsulate contacts with air. However, glass cover is not flexible enough. Our research considers a flexible CIGS PV module with ETFE cover. When the CIGS sample was exposed under radiation, the temperature of center was higher than the module margin. Because ETFE is combustible, ignition always begins at the center. The second difference is ignition time. Ignition time of a CIGS module is shorter than an Si PV module under the same conditions. The module with ETFE top layer is easier to ignite.

During the fire process, glass covers always broke into fragments. Those fragments are a potential danger for firefighters. This result would not happen with the ETFE cover layer. However, when ETFE burns, it releases hydrofluoric acid (HF). HF is extremely corrosive and toxic, therefore appropriate action must be taken when facing this condition [50]. As for different modules, the fire processes have similar single peak image of HRR. In order to evaluate fire hazard, some results can be found from Petrella’s assessment. The THRs were in the range of 38–78 MJ m^−2^ for CIGS module and 38–57 MJ m^−2^ for Si module. This means these two modules both are at intermediate risk of heat combustion. However, for the index to evaluate the risk of flashover, the glass-cover Si module had lower risk than the ETFE-cover CIGS module.

## 5. Conclusions

Research was carried out on the combustion behavior and fire hazard of CIGS thin-film solar modules. Cone calorimeter tests were conducted under five different external radiations varied from 25 to 45 kW m^−2^. Parameters were measured, such as surface temperature, ignition time, heat release rate, mass loss rate, CO concentration and CO_2_ concentration. SEM images of CIGS layers before and after burning test show that the CIGS cells structure is not changed. When the specimen was exposed to heat flux, a temperature gradient was observed. It shows the thermally thick property of the PV module. The ETFE cover layer is easily ignited when heat flux is greater than 25 kW m^−2^, which is representative of a real fire. ETFE released hydrofluoric acid during its burning process. This is a great danger for fire fighters and needs attention. The gasification heat of the module is 12.52 kJ g^−1^, and the net flame heat flux is 4.91 kW m^−2^. These two parameters are required in fire modeling.

In order to discuss the hazardous nature of fire smoke toxicity, Petrella’s evaluation system was introduced. In this research, the heat contribution risk was intermediate low with a THR range of 38–78 MJ m^−2^. The flashover risk was high when external heat flux was greater than 35 kW m^−2^. Correction calculation of FED and *LC*_50_ of under well-ventilation condition was used. *LC*_50_ was 26.72 g m^−3^ at 30 kW m^−2^, and was higher than other conditions.

With the aim of reducing the fire risk of the whole module, the barrier and encapsulant layers need more research. The flame retardancy of the front barrier determines the difficulty of ignition. Additionally, the flex backsheet and encapsulant are all combustible components. The total heat release is mainly determined by these three layers.

Different materials of cover layer or other structure in the product could lead to different hazard levels. Most studies in the thin-film solar cells area focus on electric parameters such as light transmittance, efficiency etc. Only few researches consider the PV module as a whole to study fire behavior and fire hazards. These experimental data provide basic parameters to assess PV fire. For deeper research, full-scale tests and fire propagation tests could be considered in future studies.

## Figures and Tables

**Table 1:** Petrella’s evaluation system.

Values	Total Heat Release (THR)	*x* Parameter
0.1–1.0	Very low risk to heat contribution	Low risk to flashover
1.0–10	Low risk to heat contribution	Intermediate risk to flashover
10–100	Intermediate low risk to heat contribution	High risk to flashover
100–1000	High risk to heat contribution	-

**Table 2:** Test results and thermal hazard classification.

External Heat Flux (kW m^−2^)	Peak Time (s)	pkHRR (kW m^−2^)	THR (MJ m^−2^)	*x* Parameter (kW m^−2^ s^−1^)
25	203	475	38.95 (Intermediate risk)	3.71 (Intermediate risk)
30	144	635	59.74 (Intermediate risk)	6.90 (Intermediate risk)
35	112	762	62.5 (Intermediate risk)	11.72 (High risk)
40	102	941	73.71 (Intermediate risk)	18.82 (High risk)
45	90	1024	77.72 (Intermediate risk)	26.95 (High risk)

**Table 3:** Results of gas concentration and toxicity index.

External Heat Flux (kW m^−2^)	*pk[CO]* (ppm)	*pk*[*CO*_2_] (%)	FED (-)	FED_cor_ (-)	*LC*_50_ (g m^−3^)
25	321.8	0.89	0.064	0.123	24.25
30	263.4	1.04	0.053	0.112	26.72
35	326.6	1.17	0.065	0.131	22.92
40	370.9	1.42	0.074	0.167	17.95
45	411.5	1.72	0.082	0.190	15.82
